# Decoding the complete mitochondrial genome of *Hydrangea chinensis* maxim.: insights into genomic recombination, gene transfer, and RNA editing dynamics

**DOI:** 10.1186/s12870-025-07119-z

**Published:** 2025-08-16

**Authors:** Kang Ye, Jun Qin, Hu Yonghong

**Affiliations:** Shanghai Chenshan Botanical Garden, Songjiang, Shanghai, 201602 China

**Keywords:** *Hydrangea chinensis* maxim., Mitochondrial genome, RNA editing, Gene transfer, MTPT

## Abstract

**Background:**

The mitochondrial genome (mitogenome) of plants exhibits remarkable structural complexity and evolutionary plasticity, yet remains poorly characterized in many ornamental species. *Hydrangea chinensis* Maxim. (*H. chinensis*), an indigenous plant distributed across China, serves as a vital genetic resource owing to its morphological diversity, ecological adaptability, and utility in hybrid breeding programs. Despite its importance for enhancing hydrangea varieties, no genomic resources have been published for this species to date. To bridge this knowledge gap, we sequenced and assembled the complete mitogenome of *H. chinensis*, providing novel insights into its structure, evolution, and functional dynamics.

**Results:**

The mitochondrial genome of *H. chinensis* was successfully assembled using a hybrid sequencing approach integrating long-read and short-read technologies. The mitogenome spans 722,918 base pairs with a GC content of 45.33%, encoding 38 protein-coding genes along with 21 tRNA genes and 3 rRNA genes. Comprehensive analyses revealed extensive structural features including 335 repeat pairs, 211 simple sequence repeats (SSRs), and significant recombination events that contribute to its multi-branched architecture comprising both circular and linear contigs. We identified 613 C-to-U RNA editing sites affecting key mitochondrial genes such as *nad4*, suggesting functional roles in post-transcriptional regulation. Furthermore, the genome harbors 23 plastid-derived DNA fragments (MTPTs) spanning 11,059 bp—evidence of chloroplast-to-mitochondrial gene transfer with potential evolutionary implications. Phylogenetic analysis based on conserved mitochondrial protein-coding genes positioned *H. chinensis* within the Cornales order under Hydrangeaceae while revealing extensive collinearity variations among closely related species.

**Conclusion:**

This study represents the inaugural comprehensive investigation of the mitogenome of *H. chinensis*, unveiling its intricate architecture shaped by repetitive sequences, dynamic recombination events, RNA editing processes, and inter-organelle gene transfers. These findings enhance our understanding of mitochondrial genome evolution and offer essential genetic resources to support future breeding strategies for hydrangea improvement.

**Supplementary Information:**

The online version contains supplementary material available at 10.1186/s12870-025-07119-z.

## Introduction

The *Hydrangea chinensis*, indigenous to China and Japan, is a close relative of the widely cultivated ornamental plant, the bigleaf hydrangea (*Hydrangea macrophylla* (Thunb.) Ser.), both belonging to the family Hydrangeaceae. Although *H. chinensis* is not widely cultivated as an ornamental plant, it plays a crucial role as a parent species in hybrid breeding programs aimed at improving existing hydrangea varieties. This species exhibits a broad distribution across China and demonstrates remarkable morphological diversity and adaptability, making it a valuable genetic resource for breeding efforts. To date, neither the nuclear genome nor the organelle genomes (chloroplast and mitochondrial) of H. chinensis have been sequenced, despite its widespread use in hybridization studies. Previous studies have only focused on the nuclear and mitochondrial genomes of closely related species, such as *H. macrophylla* [1]. The lack of genomic information for *H. chinensis* limits our understanding of its evolutionary dynamics and potential contributions to the genetic improvement of cultivated hydrangeas. Beyond its role in breeding, hydrangeas, including *H. chinensis*, hold substantial importance within the floriculture industry, with applications spanning landscape design, potted plants, and the cut flower markets [2]. Furthermore, owing to its purported properties such as heat-clearing, detoxification, and anti-inflammatory effects [3, 4], *H. chinensis* finds a place in traditional medicine practices.

Mitochondria serve as the cellular powerhouses and are vital for the life processes of eukaryotic organisms. Although mitochondria possess their own genomes, most mitochondrial proteins are encoded by nuclear DNA [5]. Distinctive from animal mitochondrial genomes, plant mitochondrial genomes exhibit notable variations in size and structure; they tend to be larger and more complex [6]. Typically existing as double-stranded DNA molecules that may be circular or linear depending on species specifics [7], these genomes are characterized by significant amounts of repetitive sequences. Such complexity poses challenges for assembly and sequencing due to frequent recombination events occurring within them [8]. The mutation rate of mitochondrial genomes is relatively high partly because they lack protective histones alongside efficient DNA repair mechanisms [9]. Recent advancements in long-read sequencing technologies like PacBio and Oxford Nanopore have made sequencing plant mitochondrial genomes increasingly feasible and cost-effective [10, 11].

Despite considerable focus on the nuclear genome and chloroplast genome of hydrangeas in recent years, research concerning their mitochondrial genome remains sparse. To date, there has been no systematic investigation into the mitochondrial genomic resources of hydrangeas—an oversight that constrains our understanding of evolutionary dynamics among hydrangea species’ mitochondrial genomes. Homologous recombination is a prevalent phenomenon within plant mitochondria often leading to genomic rearrangements along with duplication or exchange across intergenic regions [12]. This recombination offers novel insights into evolutionary pathways—highlighting genome diversity along with hybrid vigor aspects [13].

RNA editing—particularly C-to-U transitions—is commonly observed within plant mitochondria; this process modifies transcript coding sequences creating novel start or stop codons ultimately affecting protein function via altered amino acid sequences [14, 15]. In hydrangea’s mitochondrial genome contextually speaking RNA editing could play an instrumental role regulating both functional dynamics alongside gene expression levels though current research exploring these aspects remains limited.

Another critical genetic event is cytoplasmic inheritance where chloroplast-derived DNA segments (termed as mitochondrially located plastid DNA segments or MTPTs) integrate into the mitochondrial genome adding layers of structural complexity while hinting at potential mutational activities therein [16, 17]. Additionally cross-organelle DNA transfers unveil intriguing perspectives regarding plant genomic evolution.

In this study we sequenced t nd subsequently assembled the complete mitochondrial genome of *H. chinensis*. Our objective was not merely to elucidate its complex architecture but also to examine patterns of repetitive sequences and to provide a comprehensive analysis of recombination events throughout the genome. Moreover comparative assessments were conducted against other closely related Hydrangeaceae members focusing particularly upon inter-genomic synteny analyses amongst near-relatives which collectively enriches existing understanding surrounding evolutionary trajectories inherent within these organisms’ organellar compositions whilst furnishing invaluable genetic resources pivotal for future breeding endeavors targeting improved strains thereof [18]. By elucidating structural-functional paradigms intrinsic underlying mechanisms governing Hydrageae’s mitogenomic fabrications we aspire laying solid groundwork indispensable toward fostering continued exploration regarding organelle-centered genomic evolutions catalyzing diversity manifestations consequently impacting hybridization phenomena amidst flora at large thus paving way forward toward uncharted scientific frontiers burgeoning from foundational discoveries herein articulated.

## Materials and methods

### Plant material, DNA and RNA extraction, and sequencing

Fresh young leaves were collected from the Shuangxi Town in Pingnan County, Fujian Province (E119°03’19’’, N27°03’28”, elevation 924 m) by the Shanghai Chenshan Botanical Garden. The live leaf samples were washed with sterile water and cleaned before being flash-frozen in liquid nitrogen. Subsequently, they were stored at − 80 °C until further use. Total genomic DNA was extracted using the cetyltrimethylammonium bromide (CTAB) method [19], followed by a plant genomic DNA kit provided by Tiangen Biotech Co., Ltd., Beijing, China. The same DNA samples were utilized for sequencing on both the BGI platform and Oxford Nanopore technology. As part of this study, 350 bp fragments were incorporated into DNA libraries for short-read sequencing on the MGI BGI T7 platform. For Oxford Nanopore sequencing, DNA libraries were prepared using a kit with catalogue number SQK-LSK110, and sequencing was conducted by Wuhan Benagen Technology Co., Ltd.

### Mitochondrial genome assembly

To acquire the complete mitochondrial genome sequence of the *H. chinensis* and explore its potential structural features, this study employed a hybrid assembly method utilizing both long-read data from Oxford Nanopore sequencing and short-read data from BGI sequencing. Initially, assembly was performed directly on the long-read sequencing data using Flye (v2.9.2) software [20] with default parameters, resulting in a graphical assembly output in GFA format. All assembled contigs in FASTA format were used to construct a database with makeblastdb. Subsequently, BLASTn (v2.13.0) was employed to identify contig fragments containing mitochondrial genes using those from Arabidopsis as query sequences, with parameters set at “-evalue 1e-5 -outfmt 6 -max_hsps 10 -word_size 7 -task blastn-short.” The GFA files were visualized using Bandage software (v0.8.1) [21], enabling the screening of mitochondrial contigs based on BLASTn results to obtain a preliminary sketch of the hydrangea’s mitochondrial genome. Next, bwa software (v0.7.17) [22] was utilized to align both long-read and short-read data against the mitochondrial contigs. Aligned mitochondrial reads were filtered and exported for further use in subsequent hybrid assembly processes. Finally, employing a hybrid assembly strategy that integrates both aforementioned sequencing datasets, the hydrangea’s mitochondrial genome was assembled using Unicycler (v0.5.0) [23] with default settings, culminating in the finalized mitochondrial genome sequence. Visualization of this genome was accomplished through Bandage software.

### Annotation and codon usage bias analysis of the mitochondrial genome

To achieve a high-quality structural annotation of the mitochondrial genome, we employed Arabidopsis thaliana (NC_037304) and Liriodendron tulipifera (NC_021152.1) as reference genomes. The annotation process was conducted using Geseq software (v2.03) [24]. Additionally, the mitochondrial genome of the species was annotated using the IPMGA tool for angiosperm mitochondrial genomes (http://www.1kmpg.cn/ipmga/), which excels at annotating splice sites and trans-splicing genes. Transfer RNA genes within the mitochondrial genome were annotated with tRNAscan-SE software (v2.0.11) [25], while ribosomal RNA genes were identified using BLASTN software (v2.13.0) [26]. To enhance annotation accuracy, manual corrections and validation of potential annotation errors were performed using Apollo software (v1.11.8) [27].

For analyzing codon usage bias in mitochondrial proteins, coding sequences from the genome were first extracted utilizing Phylosuite software (v1.1.16) [28]. Subsequently, Mega software (v7.0) [29] was employed to analyze codon usage bias within protein-coding genes of the mitochondrial genome and calculate Relative Synonymous Codon Usage (RSCU) values.

### Analysis of repetitive sequences

The identification of repetitive sequences within the mitochondrial genome was conducted using a suite of computational tools. We utilized MISA (v2.1) (https://webblast.ipk-gatersleben.de/misa/) [30], TRF (Tandem Repeats Finder, v4.09) (https://tandem.bu.edu/trf/trf.unix.help.html) [31], and the REPuter web server (https://bibiserv.cebitec.uni-bielefeld.de/reputer/) [32] to detect various types of repeats, including microsatellite repeats, tandem repeats, and dispersed repeats. The visualization of these results was accomplished using Excel 2021 and the Circos package (v0.69.9) [33].

### Predictive analysis of mitochondrial chloroplast DNA

Throughout the course of mitochondrial evolution, segments of chloroplast DNA can occasionally migrate into the mitochondrial genome. The length and sequence similarity of these migrated fragments exhibit variability across different species. In this study, we employed GetOrganelle (v1.7.7.1) software [34] for the assembly of chloroplast genomes and annotated these genomes using CPGAVAS2 (v1.0) [35]. Subsequently, the annotation results were refined with the assistance of CPGView (v1.0) software [36]. A homologous segment analysis was performed using BLASTN, employing parameters set to an e-value threshold of 1e-5 and a minimum percentage identity of 80% (v2.13.0) [26], and data visualization was achieved through the Circos package (v0.69.9) [33]. This approach ultimately enabled us to identify potential migratory sequences between chloroplasts and mitochondria.

### Phylogenetic analysis

To elucidate the evolutionary history and phylogenetic position of *H. chinensis*, we selected 28 species (*Hydrangea macrophylla* (PP542010.1), *Camellia sinensis* (NC_043914.1), *Camellia gigantocarpa* (OP270590.1), *Rhododendron decorum* (NC_073150.1), *Camellia nitidissima* (NC_067639.1), Rhododendron × *pulchrum* (NC_067943.1), *Actinidia chinensis* (NC_065997.1), *Diospyros oleifera* (NC_065039.1), *Actinidia eriantha* (MZ959063.1), *Aegiceras corniculatum* (NC_056358.1), *Rhododendron simsii* (NC_053763.1), *Vaccinium macrocarpon* (NC_023338.1), *Pereskia aculeata* (NC_067638.1), *Suaeda glauca* (NC_060419.1), *Agrostemma githago* (NC_057604.1), *Mirabilis jalapa* (NC_056991.1), *Tetragonia tetragonoides* (MW971440.1), *Fallopia aubertii* (MW664926.1), *Bougainvillea spectabilis* (NC_056281.1), *Mirabilis himalaica* (NC_048974.1), *Sesuvium portulacastrum* (MN683736.1), *Chenopodium quinoa* (NC_041093.1), *Spinacia oleracea* (NC_035618.1), *Beta macrocarpa* (NC_015994.1), *Beta vulgaris* subsp. *maritima* (NC_015099.1), *Silene latifolia* (NC_014487.1), *Malania oleifera* (NC_053625.1), *Tolypanthus maclurei* (NC_056836.1)) from four orders within the angiosperms that are closely related. The mitochondrial genomes of these species were downloaded from the NCBI database (https://www.ncbi.nlm.nih.gov/genome/browse/#!/organelles/). Utilizing PhyloSuite software (v1.1.16) [28], we extracted shared genes across these species. Subsequent multiple sequence alignment was conducted with MAFFT software (v7.505) [37]. A phylogenetic tree was then constructed using the maximum likelihood method via IQ-TREE software (v1.6.12) [38], employing the parameters “--alrt 1000 -B 1000” to ensure robustness and reliability in branch support values. The Best-fit substitution model used for ML tree construction was GTR + F + I + R2, which was selected based on model testing implemented in IQ-TREE. The results of this phylogenetic analysis were visualized with ITOL software (v6) [39].

### RNA editing site identification

To identify RNA editing sites, the sequences of all protein-coding genes (PCGs) encoded by the mitochondrial genome of the species were used as input files. The tool Deepred-mt (v1.0) [40] was employed to predict C-to-U RNA editing sites within these mitochondrial PCGs. This tool leverages a convolutional neural network (CNN) model for its predictions, offering superior accuracy compared to traditional prediction methodologies. Only results with probability values exceeding 0.9 were retained, ensuring a high level of confidence in the identified editing sites.

### Synteny analysis

To identify conserved homologous sequences, known as syntenic blocks, the BLASTn program was utilized with parameters set to ‘-evalue 1e-5, -word_size 9, -gapopen 5, -gapextend 2, -reward 2, -penalty − 3’. Only syntenic blocks exceeding 500 bp in length were selected for subsequent analysis. Employing MCScanX (v1.0) [41], pairwise comparisons were conducted to generate a multiple synteny plot based on the results obtained from the BLASTn analyses. This facilitated the illustration of conserved syntenic regions. Leveraging sequence similarity data, we employed the core functionalities of MCScanX to visualize a multiple synteny plot between *H. chinensis* and its closely related species, enhancing interpretability through graphical representation.

## Result

### Assembly of the mitochondrial genome

**Fig. 1:**
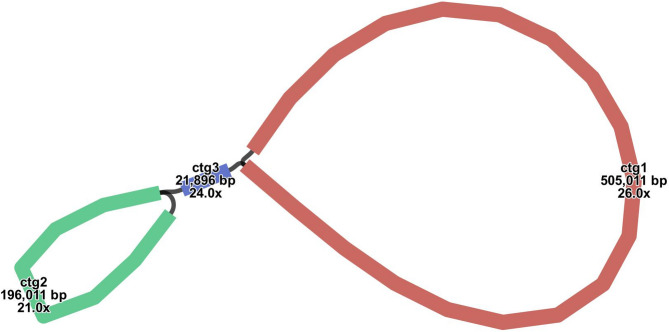
Schematic representation of the mitochondrial genome assembly of *H. chinensis*. This figure presents a visualization of the assembled mitochondrial genome, rendered using Bandage. The assembly reveals three distinct chromosomes, providing insights into the structural organization and genomic architecture of the mitogenome

To assemble the mitochondrial genome of *H. chinensis*, we employed a hybrid assembly approach that integrates both long-read and short-read sequencing data. From the total genomic DNA, we successfully assembled the *H. chinensis*’s mitochondrial genome. The preliminary draft of this assembly, constructed using long-read data, was visualized with Bandage software. This visualization yielded a graphical representation of the mitochondrial genome structure, as depicted in Fig. 1, revealing an interconnected model comprising three contigs. The principal architecture of the hydrangea mitochondrial genome is characterized by a multi-branched configuration, extending to a total length of 722,918 base pairs (bp) with a GC content of 45.33%. Detailed analysis using long-reads at junctions determined that two of these contigs can form circular structures autonomously, while one remains as an independent linear sequence (Fig. 1). Comprehensive details regarding each assembled node are summarized in Table 1. To assess the reliability of our assembly, the original reads were aligned to the assembled contigs. The coverage results (Fig S1−3) indicated continuity within the genomic sequence, with no gaps detected. This confirms the robustness and reliability of our mitochondrial genome assembly results.

**Table 1:** Summary of basic information on the *H. chinensis* mitochondrial genome

Number	Contigs	type	Length (bp)	GC content (%)
1	Chromosome 1–3	Branched	722,918	45.33
2	Chromosome 1	Circular	505,011	45.31
3	Chromosome 2	Circular	196,011	45.38
4	Chromosome 3	Linear	21,896	45.58

### Features of the mitochondrial genome

**Fig. 2:**
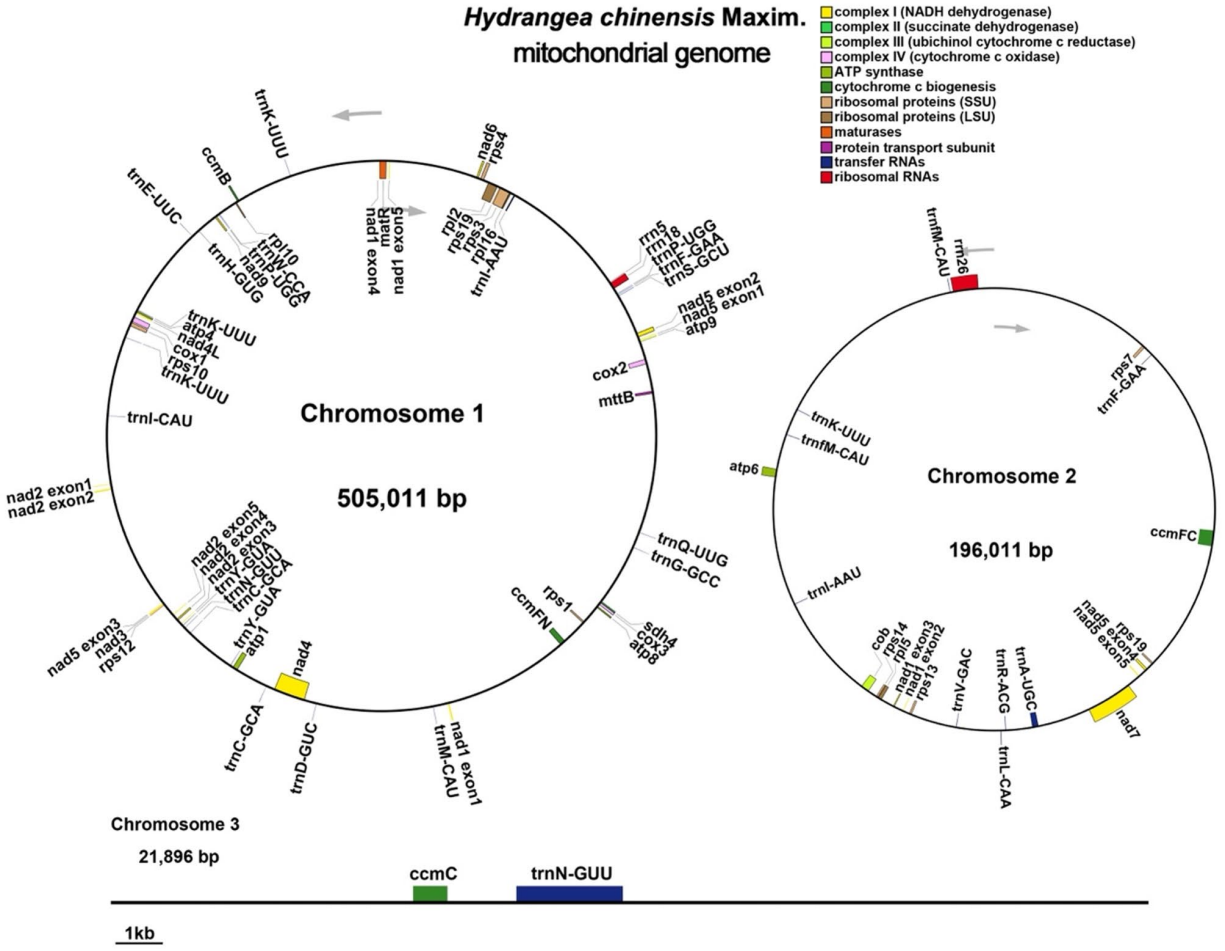
*H. chinensis* mitochondrial genome and gene annotation map. The mitochondrial genome is depicted as a circular map illustrating gene organization and transcriptional orientation, with arrows indicating the direction of transcription. Genes are color-coded according to their functional categories: protein-coding genes, tRNA genes, rRNA genes, and others. The map highlights the structural complexity of the mitogenome while providing an overview of its annotated elements

The mitochondrial genome of *H. chinensis* was meticulously annotated, resulting in the identification of 37 unique protein-coding genes, with the exception of the *rps19* gene, which has two copies. This annotation encompasses 24 distinct core mitochondrial genes and 14 non-core genes, as well as 21 tRNA genes (with eight being multi-copy) and three rRNA genes (Fig. 2). Among the core genes, we identified five ATP synthase genes (*atp1*, *atp4*, *atp6*, *atp8*, and *atp9*); nine NADH dehydrogenase subunit genes (*nad1*, *nad2*, *nad3*, *nad4*, *nad4L*, *nad5*, *nad6*, *nad7*, and *nad9*); four cytochrome c biogenesis genes (*ccmB*, *ccmC*, *ccmFC*, and *ccmFN*); three cytochrome c oxidase subunit genes (*cox1*, *cox2* and *cox3*); a single membrane transporter protein gene (*mttB*); one maturase gene (*matR*), and one ubiquinol-cytochrome c reductase gene (*cob*). The non-core segment includes four large ribosomal subunit genes (*rpl2*, *rpl5*, *rpl10* and *rpl16*) and nine small ribosomal subunit genes (*rps1*, *rps3*, *rps4*, *rps7*, *rps10*, *rps12*, *rps13*, *rps14* and *rps19*), alongside a solitary succinate dehydrogenase gene (*sdh4*) (Table 2).

**Table 2:** Coding genes of the *H. chinensis* mitochondrial genome

Group of genes	Name of genes
ATP synthase	*atp1*, *atp4*, *atp6*, *atp8*, *atp9*
NADH dehydrogenase	*nad1*, *nad2*, *nad3*, *nad4*, *nad4L*, *nad5*, *nad6*, *nad7*, *nad9*
Cytochrome *b*	*cob*
Cytochrome *c* biogenesis	*ccmB*, *ccmC*, *ccmFC*, *ccmFN*
Cytochrome *c*	*oxidase cox1*, *cox2*, *cox3*
Maturases	*matR*
Protein transport subunit	*mttB*
Ribosomal protein large subunit	*rpl2*, *rpl5*, *rpl10*, *rpl16*
Ribosomal protein small subunit	*rps1*, *rps3*, *rps4*, *rps7*, *rps10*, *rps12*, *rps13*, *rps14*, *rps19* (×2)
Succinate dehydrogenase	*sdh4*
Ribosome RNA	*rrn5*, *rrn18*, *rrn26*
Transfer RNA	*trnA*-UGC, *trnC*-GCA(×2), *trnD*-GUC, *trnE*-UUC, *trnF*-GAA (×2), *trnfM*-CAU (×2), *trnG*-GCC, *trnH*-GUG, *trnI*-AAU (×2), *trnI*-CAU, *trnK*-UUU (×4), *trnL*-CAA, *trnM*-CAU, *trnN*-GUU (×2), *trnP*-UGG (×2), *trnQ*-UUG, *trnR*-ACG, *trnS*-GCU, *trnV*-GAC, *trnW*-CCA, *trnY*-GUA (×2)

The numbers in parentheses represent the copy number of the gene, such as (×2) indicating two copies

**Fig. 3:**
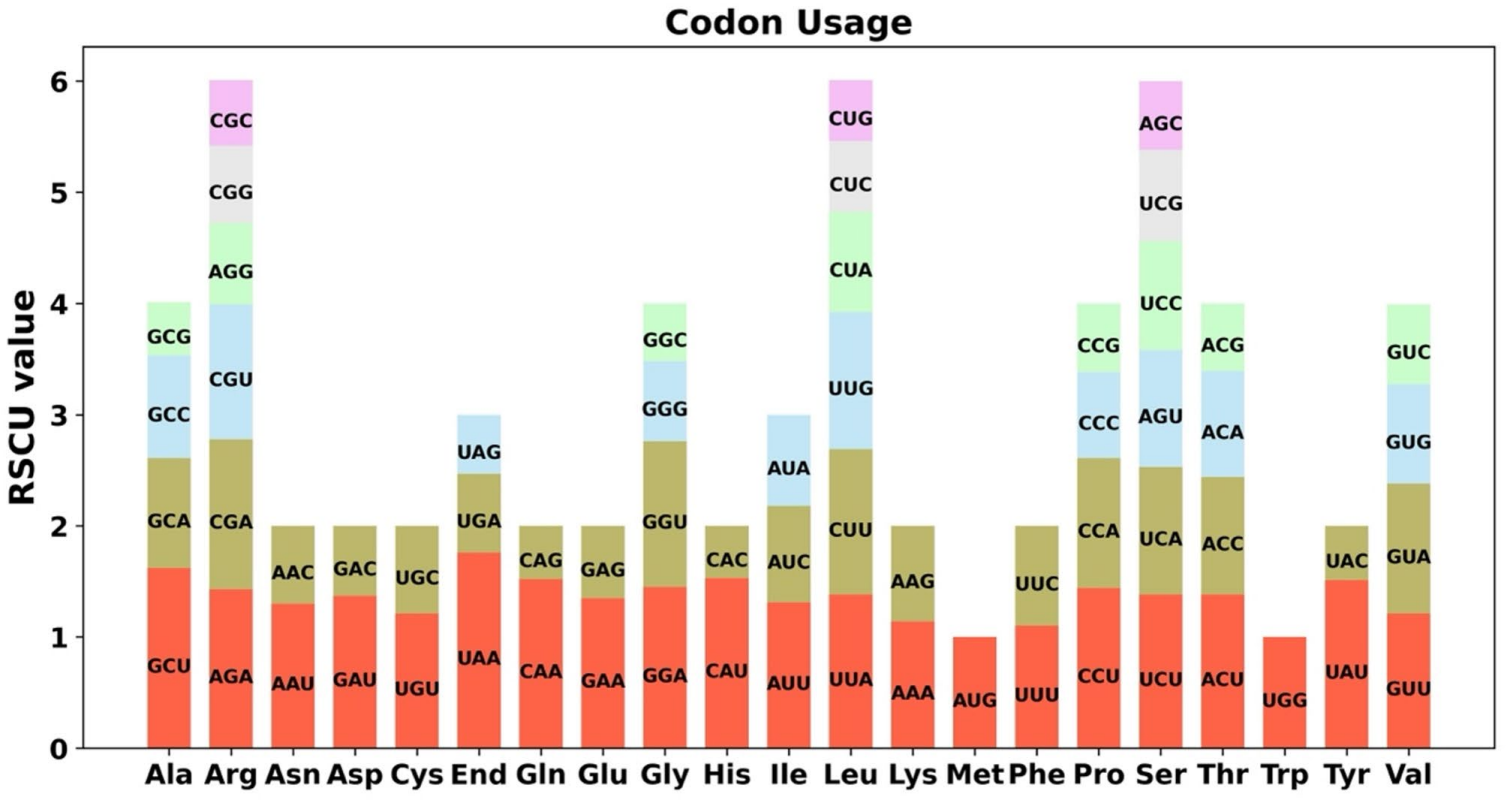
Codon usage bias analysis of the mitochondrial genome of *H. chinensis*. This figure illustrates the relative synonymous codon usage (RSCU) values for protein-coding genes within the mitochondrial genome of *H. chinensis*. The codon usage patterns are depicted for 20 standard amino acids as well as termination (stop) codons, denoted as “End.” Elevated RSCU values highlight a preferential selection for specific codons over their synonymous counterparts, shedding light on translational efficiency and potential adaptive biases in the organism’s mitochondrial genome

Codon preference refers to the differential frequency with which synonymous codons are employed during the translation process and the evolutionary establishment of a set of preferred codons that align with it, playing a crucial role in gene expression. There is a marked variation in codon usage among different species. Consequently, this study calculated the Relative Synonymous Codon Usage (RSCU) to investigate codon preference in the 38 protein-coding genes (PCGs) of the *H. chinensis* mitochondrial genome. The usage distribution for each amino acid across various codons is detailed in Table S1. Codons with an RSCU value greater than 1 are considered to be preferentially used by specific amino acids. As depicted in Fig. 3, aside from the start codon AUG and tryptophan (UGG), both of which possess an RSCU value of 1, there is an evident pattern of preferred codon usage within the mitochondrial PCGs. For instance, the stop codon UAA exhibits a strong preference, boasting the highest RSCU value within mitochondrial PCGs at 1.76. Similarly, alanine (Ala) shows a pronounced preference for GCU, with an RSCU value of 1.56. To further explore codon usage patterns and their relationship with closely related species, we conducted a Codon Usage Bias Analysis on the Mitochondrial Genome of H. macrophylla (see Table S2 and Fig S4). The comparison revealed a remarkably high consistency in codon usage preferences between these two species, indicating that codon usage exhibits substantial uniformity within congeneric species.

### Repetitive sequences in the mitochondrial genome

**Fig. 4:**
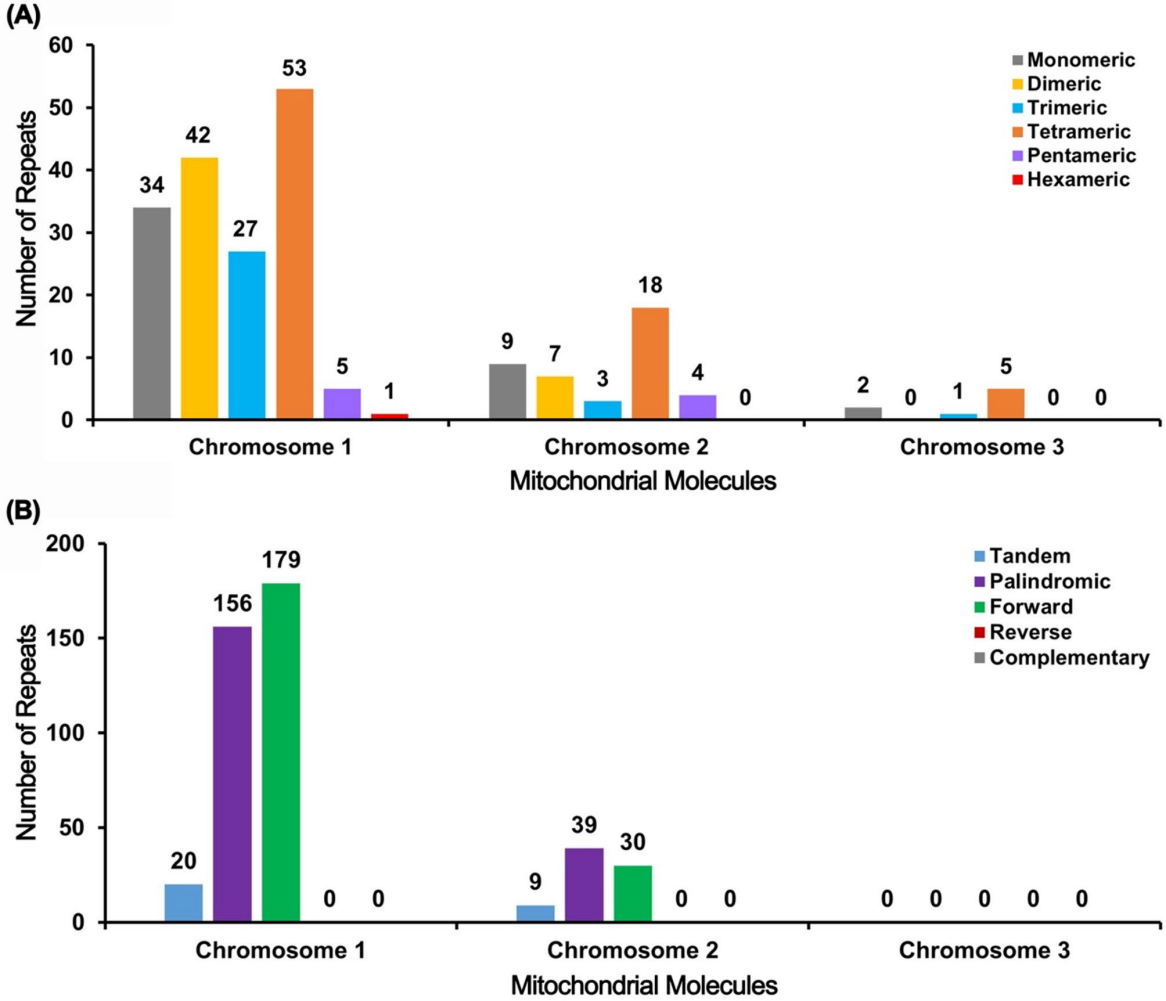
Bar Chart analysis of repeat sequences. **A** The x-axis represents mitochondrial molecules, while the y-axis denotes the number of repeat fragments. The legend is as follows: gray indicates mononucleotide SSRs, yellow represents dinucleotide SSRs, blue signifies trinucleotide SSRs, orange denotes tetranucleotide SSRs, purple symbolizes pentanucleotide SSRs, and red stands for hexanucleotide SSRs. **B** The x-axis again corresponds to mitochondrial molecules, with the y-axis depicting the number of repeat fragments. The legend is defined as: blue for tandem repeats, purple for palindromic repeats, green for forward repeats, red for reverse repeats, and gray for complementary repeats

**Fig. 5:**
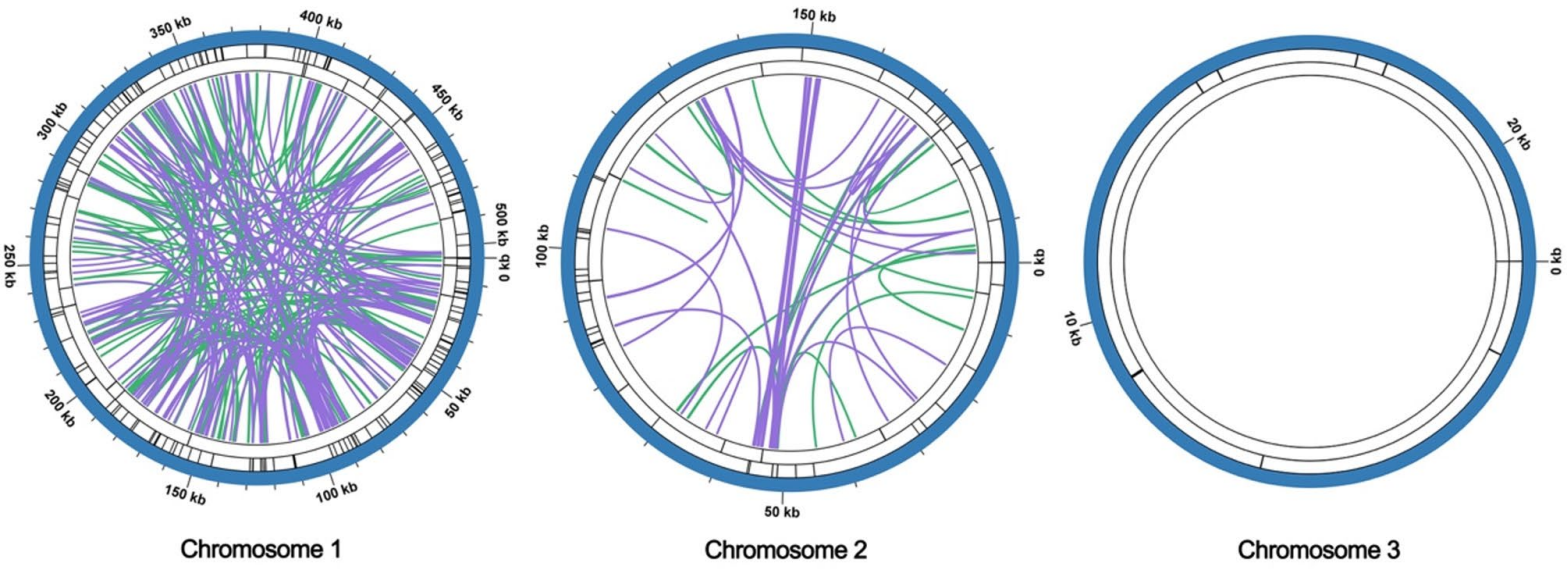
Chord diagram analysis of repeat sequences. Repeat sequence analysis was conducted for three mitochondrial molecules, with Chromosome 1 serving as an exemplar. In the innermost circle, colored lines connect the two sequences of dispersed repeats: purple lines indicate palindromic repeats, while green lines denote forward repeats. The second circle features black segments representing tandem repeat sequences, and the outermost circle contains black segments indicating microsatellite repeat sequences. This pattern continues similarly for Chromosome 2 through Chromosome 3

Repetitive sequences play a pivotal role not only in maintaining the higher-order structure of genomes but also in driving evolution, inducing variation, and regulating gene expression. Consequently, we conducted an analysis of dispersed repeats, microsatellites, and tandem repeats within the *H. chinensis* organelle genome (Fig. 4A). Across the three mitochondrial contigs, we identified 162, 41, and 8 SSRs (Simple Sequence Repeats), respectively. Tetrameric SSRs were found to be the most abundant, whereas hexameric SSRs were the least common. In Chromosome 1, there are 20 tandem repeat sequences with a match ratio exceeding 70%, ranging in length from 9 to 39 base pairs. Additionally, there are 335 repeat pairs with lengths greater than or equal to 30 base pairs; among these, palindromic repeats account for 156 pairs and forward repeats for 179 pairs. Chromosome 2 contains nine tandem repeat sequences with a match ratio above 81%, spanning lengths from 11 to 30 base pairs. This chromosome features 69 repeat pairs longer than or equal to 30 base pairs—39 palindromic and 30 forward repeats. Notably, Chromosome 3 lacks any repeat sequence pairs exceeding or equalling a length of 30 base pairs as well as any other scattered repetitive sequences (Fig. 4B). Figure 5, in particular, illustrates the detailed genomic positional distribution of various repeat sequences, such as palindromic repeats and forward repeats. Through further examination of these repeat sequences across different contigs (Figure S5), our analysis reveals that there are no substantial long segment duplications within the mitochondrial genomes of this species, with the longest repeat segment measuring merely 482 base pairs. The abundance of repetitive sequences provides crucial data for selecting molecular markers that could be used to study the genetic diversity of hydrangeas.

### Prediction of RNA editing sites

**Fig. 6:**
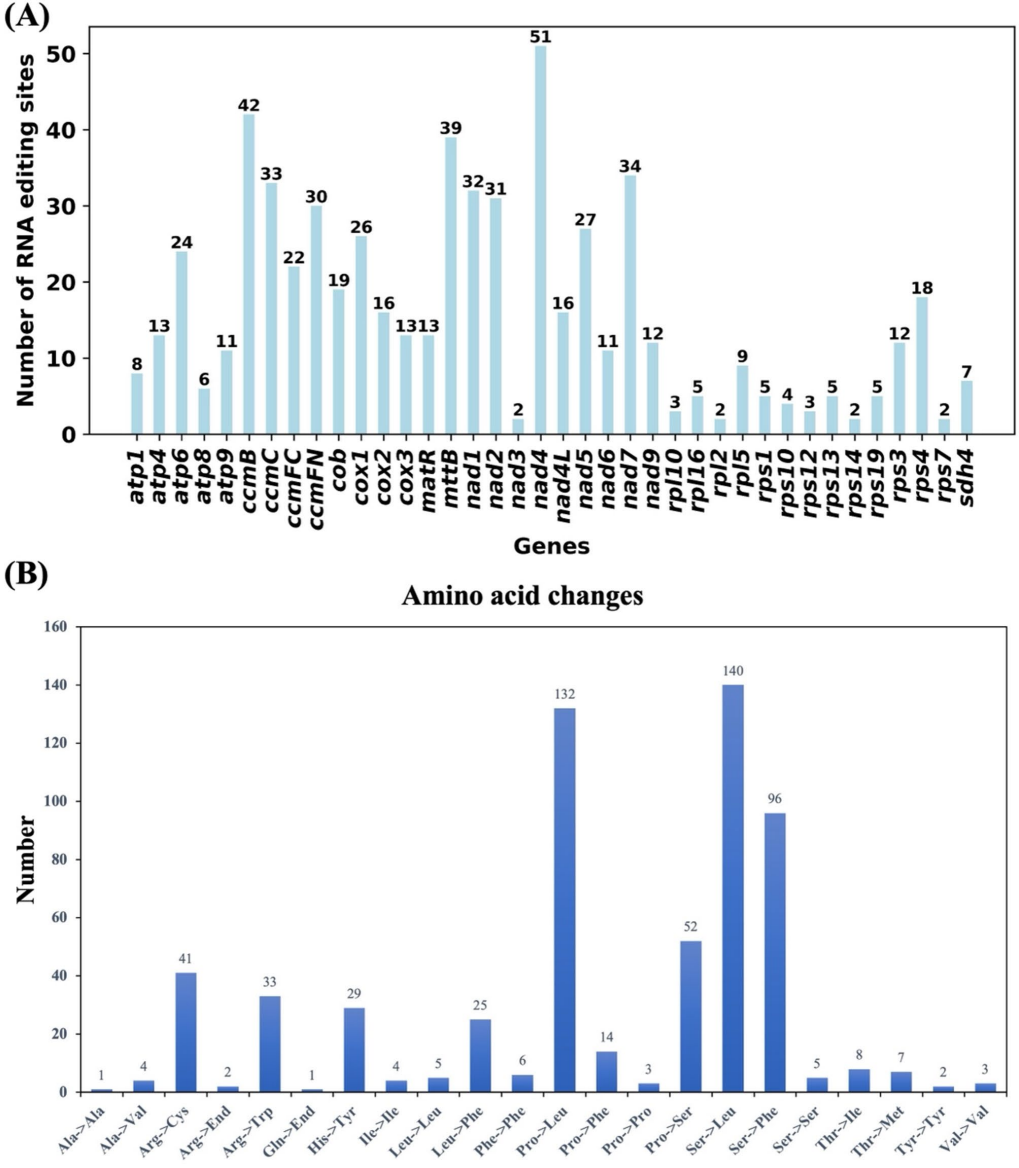
Overview of RNA editing events. **A** Distribution of predicted RNA editing sites across various mitochondrial protein-coding genes (PCGs). **B** Distribution of the number of amino acid changes

RNA editing is a process characterized by the insertion, deletion, or alteration of nucleotides during DNA transcription, occurring in mitochondria, chloroplasts, and nuclei to form RNA. Utilizing the Deepred-mt tool, we identified RNA editing events within 38 unique protein-coding genes (PCGs) from *H. chinensis* mitochondria. These genes include *atp1*, *atp4*, *atp6*, *atp8*, *atp9*, *ccmB*, *ccmC*, *ccmFC*, *ccmFN*, *cob*, *cox1*, *cox2*, *cox3*, *matR*, *mttB*, *nad1* through *nad7* and *nad9*, *rpl10* and *rpl16* through *rps7* as well as *sdh4* (Fig. 6A and Table S3). Ultimately applying a selection threshold greater than 0.9 led to the identification of 613 potential RNA editing sites—all involving C-to-U base conversions. Among the mitochondrial genes analyzed, *nad4* exhibited the highest frequency of RNA editing with 51 identified sites. This was followed by the *ccmB* gene with 42 detected editing sites. Conversely, *nad3* and genes such as *rpl2*, *rps14* and *rps7* showed minimal editing activity with merely two events each. Notably among these events were 29 synonymous mutations where nucleotide substitutions did not alter the corresponding amino acids. Furthermore, there were two instances exceeding 100 occurrences: Proline transforming into Leucine and Serine transitioning into Leucine (Fig. 6B). These RNA editing events have the potential to alter protein functionality, possibly impacting their roles in mitochondrial respiration and energy production.

### Prediction of mitochondrial plastid DNA

**Fig. 7:**
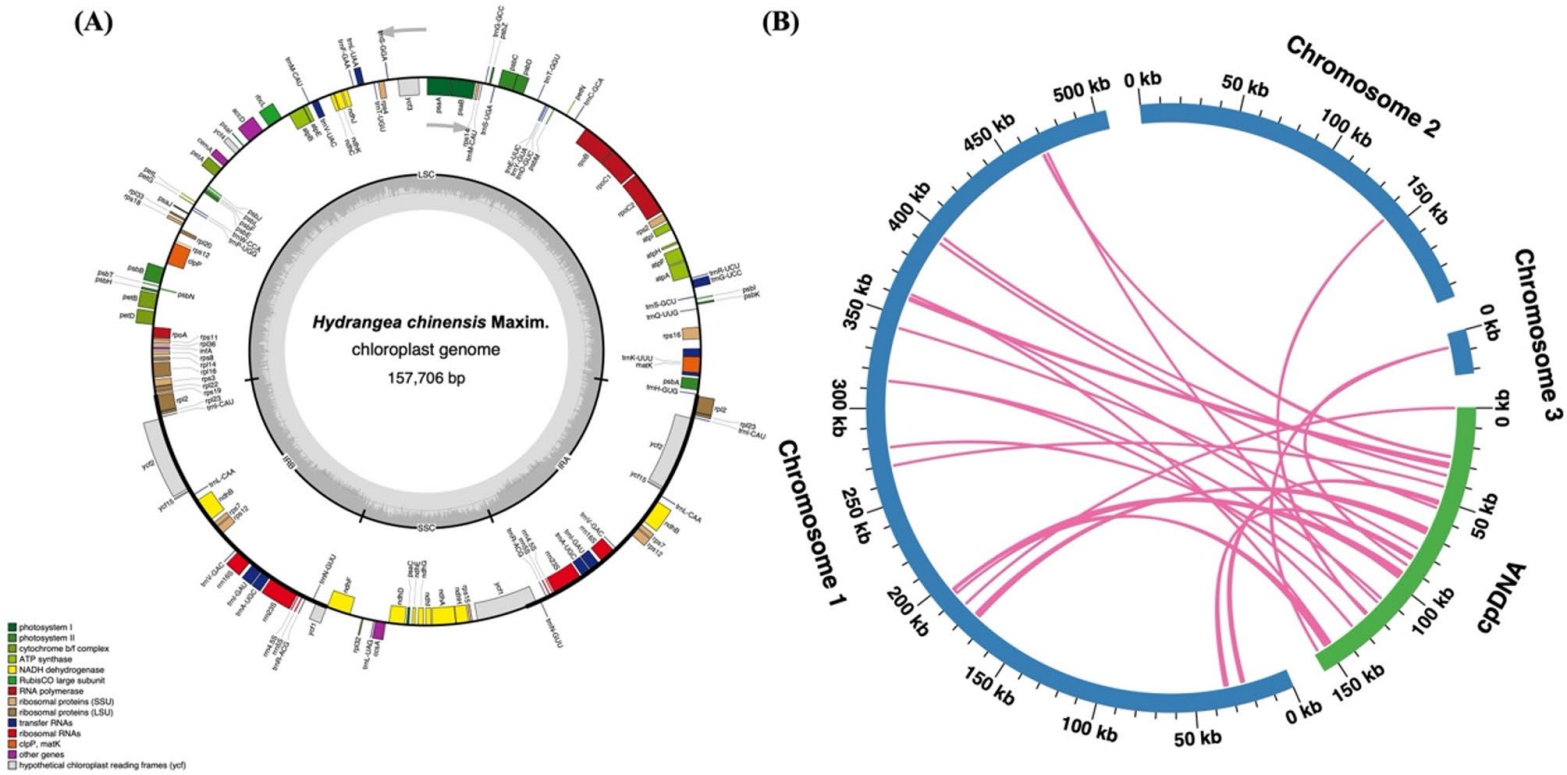
Overview of sequence transfer. **A** Chloroplast genome and gene annotation map. **B** Circos plot of sequence transfer. In the diagram, blue arcs represent the mitochondrial genome, while green arcs denote the chloroplast genome. The pink connecting lines between arcs correspond to homologous genomic segments

Mitochondrial plastid DNAs (MTPTs) are remnants of chloroplast-origin DNA within mitochondrial genomes, indicating segments originally derived from the chloroplast genome subsequently integrated into the mitochondrial genome. In this study, we employed a methodology akin to that used for mitochondria to assemble the chloroplast genome of *H. chinensis*, which spans a length of 157,706 base pairs. This assembly revealed predictions for 88 protein-coding genes, 57 tRNA genes, and 8 rRNA genes (Fig. 7A). Through sequence similarity analysis within the hydrangea species, we identified a total of 23 homologous fragments shared between the mitochondrial and chloroplast genomes, with a cumulative length of 11,059 base pairs—comprising 1.53% of the mitochondrial genome’s total length (Fig. 7B and Table S4). Among these fragments, MTPT1 emerged as the longest at 2,942 base pairs. Annotation of these homologous sequences uncovered 18 complete genes across the 23 homologous segments. These include 11 protein-coding genes (*ndhJ*, *ndhK*, *petL*, *petN*, *psbE*, *psbF*, *psbJ*, *psbL*, *psbM*, *rpl14* and *ycf15*) and an array of seven tRNA genes (*trnD*-GUC, *trnH*-GUG, *trnI*-CAU, *trnM*-CAU *trnN*-GUU *trnP*-UGG *trnW*-CCA). These findings underscore the intricate genomic interactions between the mitochondrial and chloroplast genomes, highlighting the evolutionary significance of gene transfer events in shaping the genomic architecture of hydrangea.

### Phylogenetic relationships

**Fig. 8:**
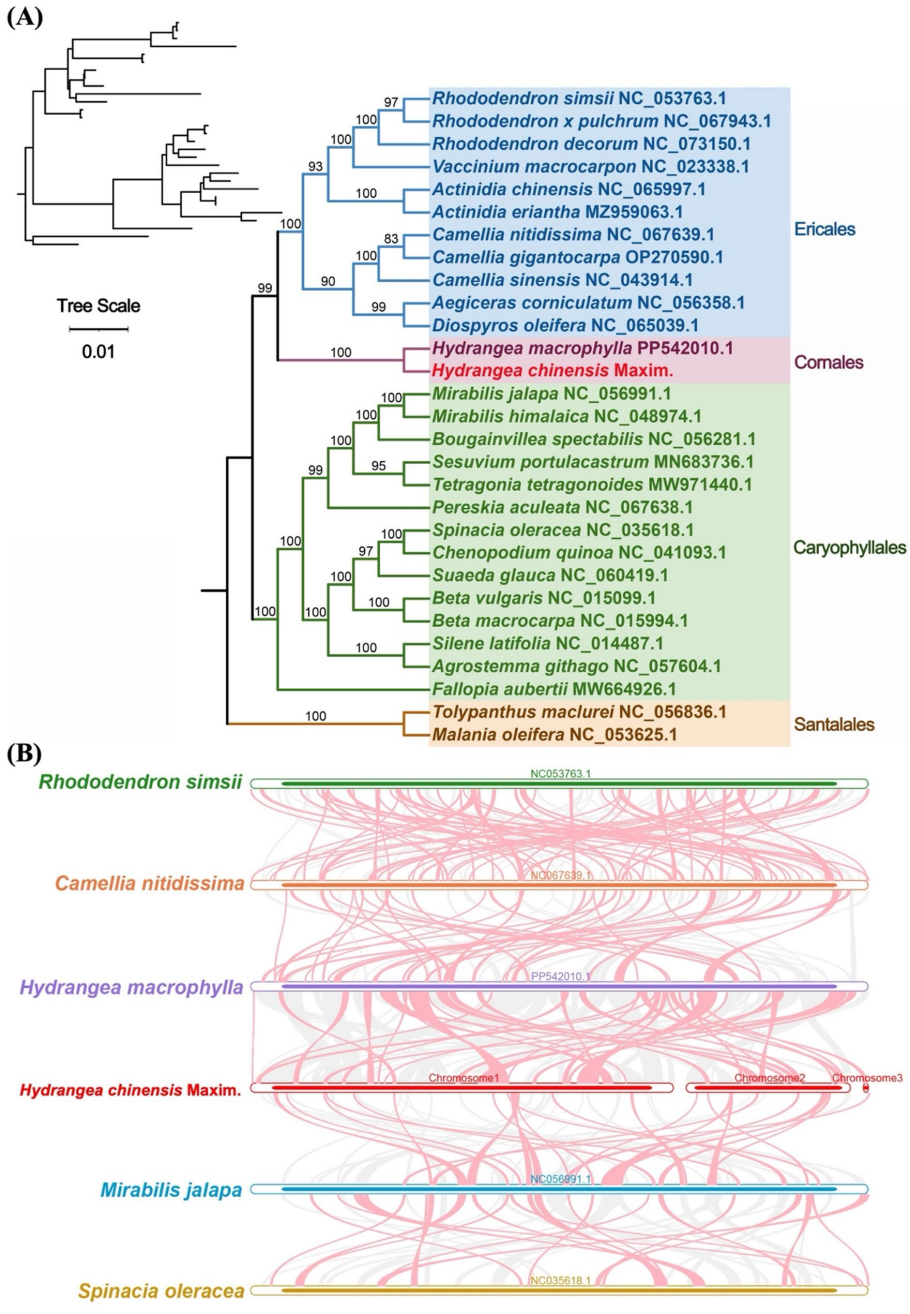
Phylogenetic Relationships and Chromosomal Recombination Events in *H. chinensis.* **A** Phylogenetic Analysis. A maximum likelihood (ML) phylogenetic tree was constructed based on 19 conserved protein-coding genes (*atp1*,* atp4*,* atp6*,* atp8*,* atp9*,* ccmB*,* ccmFC*,* ccmFN*,* cob*,* cox2*,* cox3*,* matR*,* nad1*,* nad3*,* nad5*,* nad6*,* nad7*,* rpl5*,* rps12*) from the mitochondrial genomes of 29 plant species. The numbers above the nodes represent support values, including ML bootstrap values and branch lengths. The blue region denotes Ericales, the pink region signifies Cornales, the green region represents Caryophyllales, and the orange region indicates Santalales; the Hydrangea studied is highlighted in red. **B** Syntenic Blocks Between *H. chinensis* and Five Closely Related Species. The red arc regions indicate areas where inversions have occurred, while gray regions represent areas of high homology. For clarity in presentation, syntenic blocks shorter than 0.5 kb were not included in the results

To elucidate the evolutionary history of *H. chinensis*, we constructed a phylogenetic tree using the mitochondrial genomes of 29 species across four orders of angiosperms. Initially, we identified 19 conserved mitochondrial protein-coding genes’ DNA sequences through shared gene identification: *atp1*, *atp4*, *atp6*, *atp8*, *atp9*, *ccmB*, *ccmFC*, *ccmFN*, *cob*, *cox2*, *cox3*, *matR*, *nad1*, *nad3*, *nad5*, *nad6*, *nad7*, *rpl5* and *rps12*. Using two Santalales mitochondrial genomes as outgroups allowed for the construction of a phylogenetic tree (Fig. 8A). The phylogenetic analysis demonstrated that *H. chinensis* belongs to the order Cornales within the family Hydrangeaceae and occupies a phylogenetic position between Caryophyllales and Ericales. This topology based on mitochondrial DNA aligns with the latest classification by APG (Angiosperm Phylogeny Group). The complete mitochondrial genome of *H. chinensis* represents a valuable asset for developing molecular markers and enhancing our understanding of its evolutionary history and cultivation strategies.

Further analysis of collinear regions within organelle genomes from six representative species across different families and orders revealed an abundance of homologous collinear segments. To enhance the clarity of our findings, we excluded collinear blocks shorter than 0.5 kb from the presented results (Fig. 8B). Our investigations detected homologous collinear blocks between hydrangea and its phylogenetically related species, albeit these blocks were relatively short. The findings indicate a lack of consistency in the arrangement of mitochondrial genome collinear blocks among these six species.

Remarkably, synteny between the mitochondrial genomes of hydrangeas and their closely related species is relatively poor, marked by extensive genome rearrangements and substantial structural differences. For example, a mere 214 collinear regions were discerned between *H. chinensis* and *M. jalapa*, collectively spanning just 107,839 base pairs (bp). In stark contrast, comparisons involving *H. macrophylla* and *C. nitidissima* uncovered as many as 581 collinear regions; however, their total extent reached only 207,594 bp. Among the hydrangea species themselves, the mitochondrial genome synteny between *H. chinensis* and *H. macrophylla* is comparatively better—exhibiting 414 shared segments with a total size of 494,614 bp. Nevertheless, even within different species of the Hydrangea genus, significant genomic rearrangement events are evident in their mitochondria, indicating pronounced differences remain. This suggests that these two hydrangea are genetically distinct from each other and possess evolutionary relationships that are more distant than previously anticipated.

## Discussion

### Structural complexity and recombination-driven diversity in the mitochondrial genome

The mitochondrial genome of *H. chinensis* exhibits a complex structure, characterized by three contigs, two of which can form circular configurations, while one remains linear. This multi-branched architecture is consistent with the structural plasticity observed in other plant mitochondrial genomes, which often undergo recombination and rearrangement events due to the presence of large repeat sequences and homologous recombination mechanisms [42, 43]. The identification of 335 repeat pairs, including palindromic and forward repeats, highlights the potential for frequent recombination, which may drive genomic rearrangements and contribute to the structural diversity of the mitochondrial genome. These findings align with previous studies on plant mitochondrial genomes, where recombination events have been shown to play a crucial role in genome evolution and adaptation [44]. The presence of numerous simple sequence repeats (SSRs) further underscores the dynamic nature of the mitochondrial genome. SSRs are known to contribute to genome instability and variability, which in turn can influence gene expression and mitochondrial function [45]. The abundance of SSRs in *H. chinensis* provides a valuable resource for the development of molecular markers, which could be used to study genetic diversity and phylogenetic relationships within the Hydrangea genus and across related species.

### RNA editing and its role in mitochondrial function

Our analysis of RNA editing events in the mitochondrial genome of *H. chinensis* revealed a total of 613 C-to-U editing sites, a phenomenon commonly observed in plant mitochondria [46]. These RNA editing events are known to play a critical role in post-transcriptional regulation by altering codons, which can lead to changes in amino acid sequences, the creation of new start or stop codons, and ultimately the modification of protein function [46]. In *H. chinensis*, the *nad4* gene exhibited the highest number of editing sites, suggesting that this gene may undergo extensive post-transcriptional modifications, potentially influencing its role in mitochondrial respiration and energy production. The identification of 29 synonymous mutations among the RNA editing events indicates that not all editing events result in functional changes at the protein level. However, the non-synonymous editing events observed in key mitochondrial genes, such as *cox1*, *atp6*, and *ccmB*, suggest that RNA editing may be essential for the proper functioning of the electron transport chain and other mitochondrial processes. These findings are consistent with previous studies on RNA editing in plant mitochondria, where editing has been shown to be crucial for maintaining mitochondrial integrity and function [47].

### Evidence of chloroplast-to-mitochondrial DNA transfer

The detection of 23 mitochondrial plastid DNA (MTPT) fragments in the *H. chinensis* mitochondrial genome provides evidence of gene transfer from the chloroplast genome, a process that is well-documented in plant organelles [48]. These MTPTs, which account for 1.53% of the mitochondrial genome, include both protein-coding genes and tRNA genes, suggesting that chloroplast-derived sequences may have been co-opted by the mitochondrial genome over evolutionary time. The presence of MTPTs highlights the dynamic nature of plant mitochondrial genomes, which are known to incorporate foreign DNA from various sources, including chloroplasts and even the nuclear genome [49]. The functional significance of these MTPTs remains unclear, but their integration into the mitochondrial genome may contribute to genomic rearrangements and the generation of novel genetic combinations. Additionally, the presence of chloroplast-derived tRNA genes in the mitochondrial genome may play a role in optimizing mitochondrial translation processes, although further research is needed to elucidate the exact functions of these transferred sequences.

### Phylogenetic insights and evolutionary dynamics of *H. chinensis*

Our phylogenetic analysis, based on 19 conserved mitochondrial protein-coding genes, positioned *H. chinensis* within the order Cornales, in agreement with the latest APG (Angiosperm Phylogeny Group) classification [50]. This confirms the phylogenetic placement of *H. chinensis* and provides further evidence of the evolutionary relationships between hydrangea and other members of the Cornales order. The close phylogenetic relationship between *H. chinensis* and species from the families Caryophyllaceae and Ericaceae, as revealed by collinearity analysis, suggests that these species may share common evolutionary mechanisms, particularly in terms of mitochondrial genome rearrangements and RNA editing events. Interestingly, our comparative analysis of collinear regions across six representative species revealed significant genomic rearrangements between *H. chinensis* and its phylogenetically related species, particularly in the mitochondrial genome. These findings suggest that the mitochondrial genomes of hydrangea species have undergone extensive structural divergence, possibly driven by recombination events, gene transfer, and selection pressures. The observed divergence between mitochondrial genomes of different hydrangea further emphasizes the genetic complexity and evolutionary plasticity within this genus.

## Conclusions

This study presents the first comprehensive assembly and characterization of the *H. chinensis* mitochondrial genome, providing valuable insights into its structural complexity and evolutionary dynamics. Our findings reveal extensive genomic recombination and the integration of chloroplast-derived segments, underscoring the dynamic nature of the mitogenome in this species. The identification of numerous RNA editing sites, including those affecting start and stop codons, highlights the potential for post-transcriptional regulation of mitochondrial gene expression in *H. chinensis.* Collectively, these results not only enhance our understanding of mitochondrial genome organization and evolution within the Hydrangea genus but also establish a foundational resource for future comparative genomics and molecular breeding efforts in ornamental plants.

## Supplementary Information

Below is the link to the electronic supplementary material.

Supplementary Material 1

Supplementary Material 2

## Data Availability

The datasets presented in this study can be found in online repositories. The names of the repository/repositories and accession number(s) can be found in the article/Supplementary Material. The mitogenome sequence supporting the conclusions of this article is available in GenBank (https://www.ncbi.nlm.nih.gov/) under accession number: PQ593888, PQ593889, PQ593890. The chloroplast genome sequence information has been uploaded to NCBI (accession number: PQ593891).

## Declarations

### Permission for material collection

We are licensed to collect the plant of *Hydrangea chinensis* from Shuangxi Town in Pingnan County, Fujian Province, China (E119°03’19’’, N27°03’28”, elevation 924 m). The collection was conducted by the Shanghai Chenshan Botanical Garden and complies with relevant institutional, national, and international guidelines and legislation.
