# Supplementary material for: Decoding the complete mitochondrial genome of Hydrangea chinensis maxim.: insights into genomic recombination, gene transfer, and RNA editing dynamics
Source: BMC Plant Biol. 2025 Aug 16;25:1078. doi: 10.1186/s12870-025-07119-z (PMC12357480; doi:10.1186/s12870-025-07119-z)
Supplement: Supplementary file 2 — Supplementary Material 2 [file 12870_2025_7119_MOESM2_ESM.docx]

Supplement Figures:


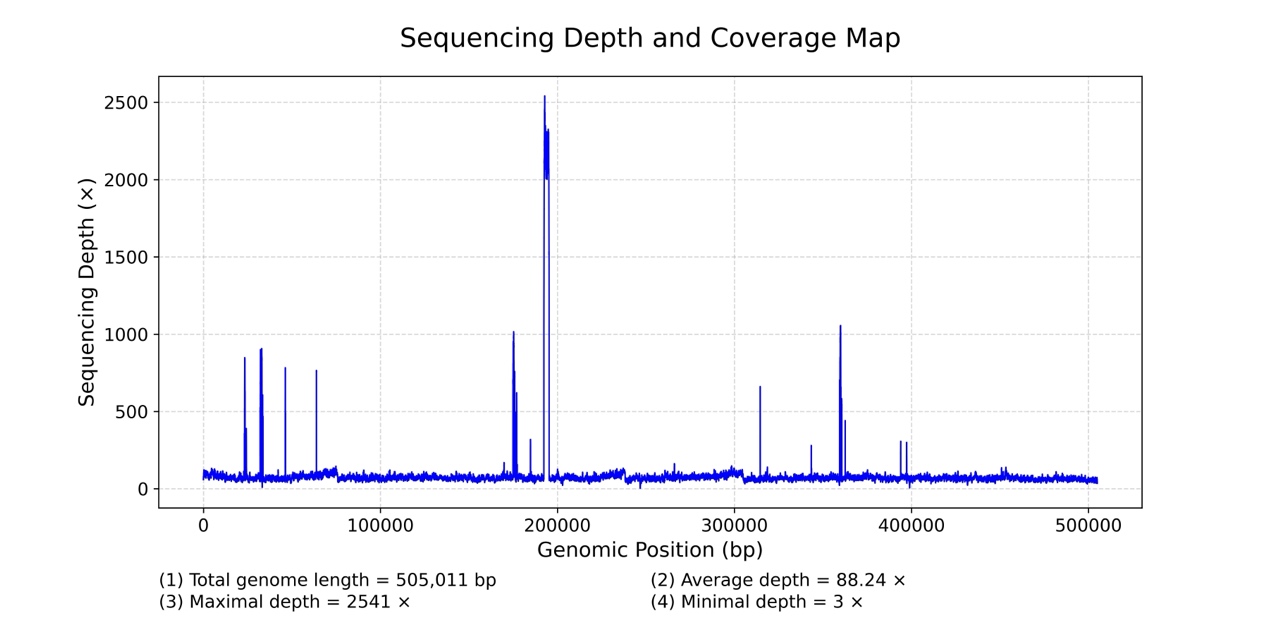


Fig S1. Coverage Distribution Graph of Contig 1: The x-axis represents the positional coordinates, while the y-axis denotes the depth of coverage.


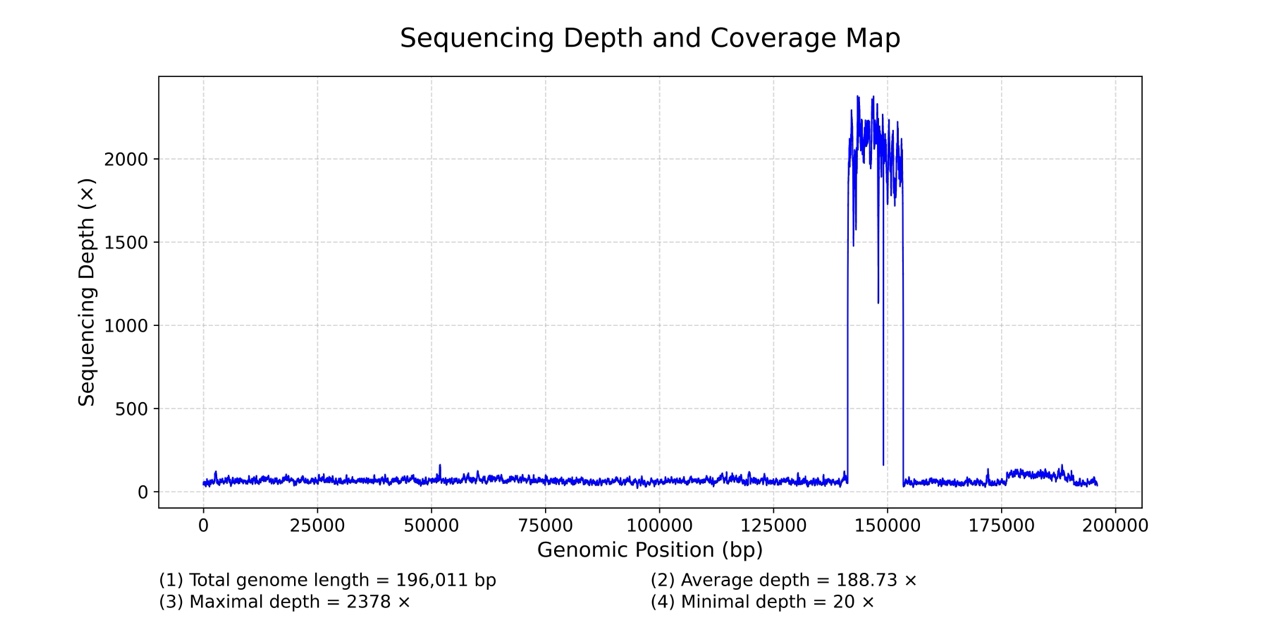


Fig S2 Coverage Distribution Graph of Contig 2: The x-axis represents the positional coordinates, while the y-axis denotes the depth of coverage.


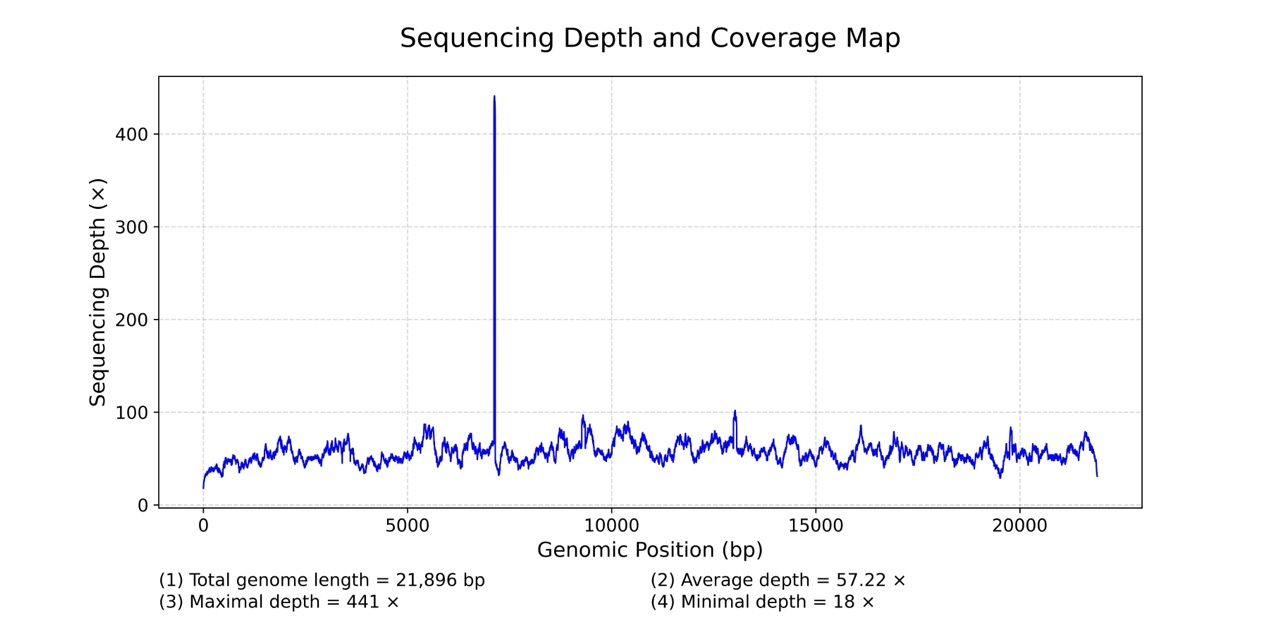


Fig S3 Coverage Distribution Graph of Contig 3: The x-axis represents the positional coordinates, while the y-axis denotes the depth of coverage.


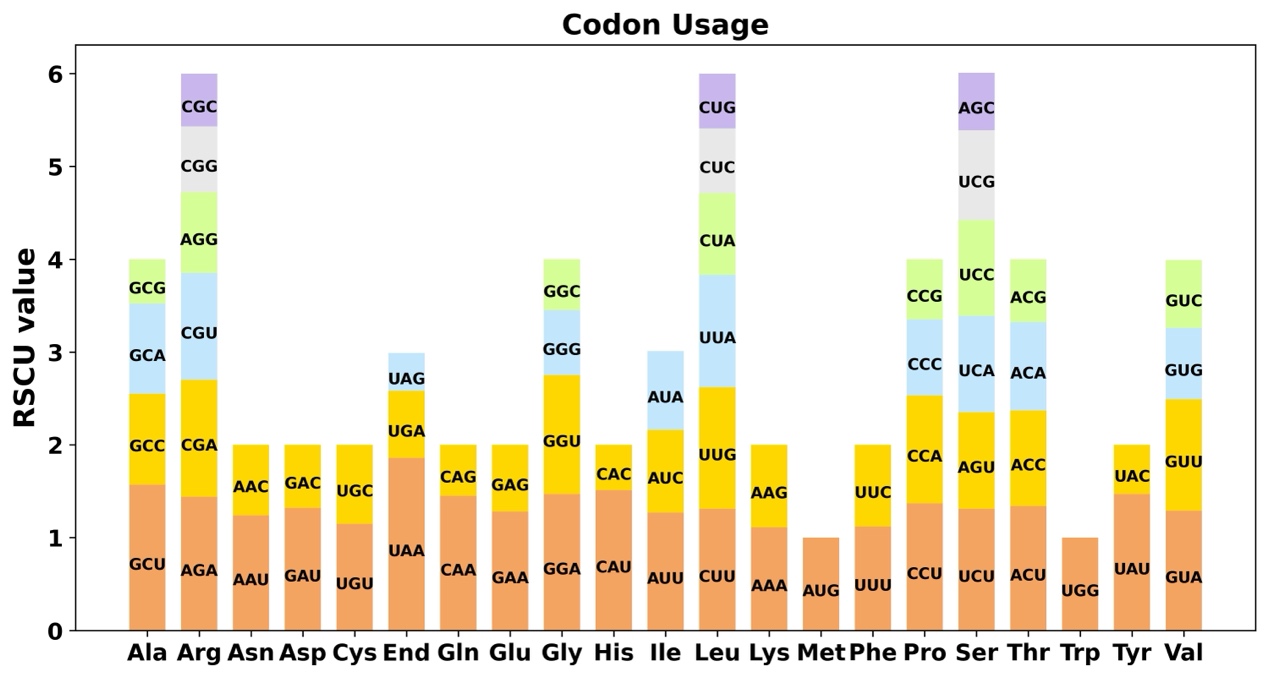


Fig S4 Codon Usage Bias Analysis of the Mitochondrial Genome of *H. macrophylla*. This figure illustrates the relative synonymous codon usage (RSCU) values for protein-coding genes within the mitochondrial genome of *H. macrophylla*. The codon usage patterns are depicted for 20 standard amino acids as well as termination (stop) codons, denoted as "End." Elevated RSCU values highlight a preferential selection for specific codons over their synonymous counterparts, shedding light on translational efficiency and potential adaptive biases in the organism's mitochondrial genome.


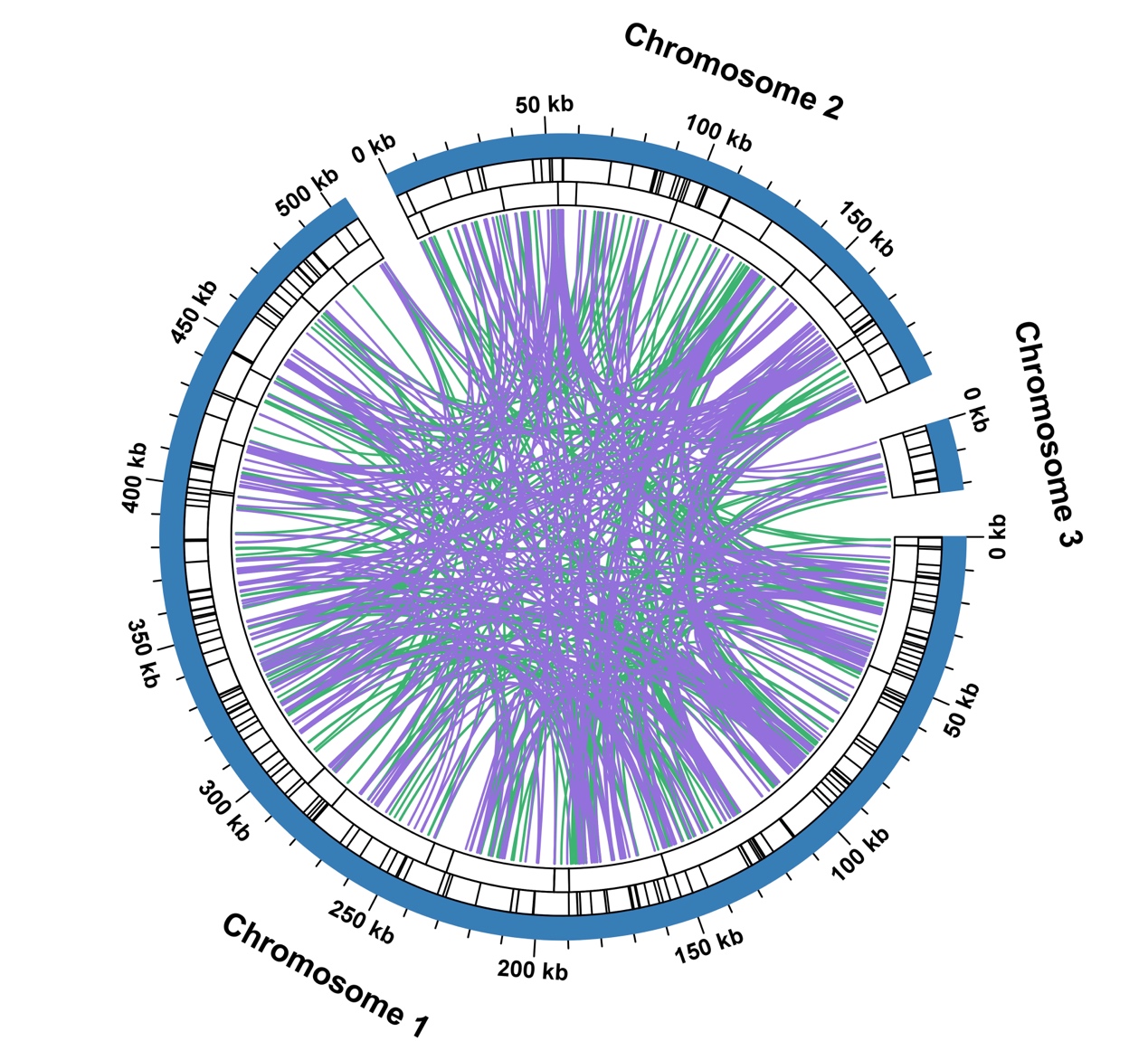


Fig S5 Chord Diagram Analysis of Repeat Sequences. Repeat sequence analysis was conducted for three mitochondrial molecules，encompassing the analysis of interchromosomal repeat sequences. In the innermost circle, colored lines connect the two sequences of dispersed repeats: purple lines indicate palindromic repeats, while green lines denote forward repeats. The second circle features black segments representing tandem repeat sequences, and the outermost circle contains black segments indicating microsatellite repeat sequences.
